# Changes in eating habits and lifestyle during the first year of the COVID‐19 pandemic across metropolitan regions in Brazil and Germany: A survey‐based cross‐sectional study

**DOI:** 10.1002/fsn3.3960

**Published:** 2024-01-22

**Authors:** Juliana M. G. Paris, Emília M. F. Lima, Jéssica de A. F. F. Finger, William R. Isidorio, Christine Heinzel, Timo Falkenberg, Christian Borgemeister, Uelinton M. Pinto, Ute Nöthlings

**Affiliations:** ^1^ Center for Development Research (ZEF) University of Bonn Bonn North‐Rhine Westphalia Germany; ^2^ Food Research Center (FoRC‐CEPID), Department of Food and Experimental Nutrition, Faculty of Pharmaceutical Sciences University of São Paulo São Paulo São Paulo Brazil; ^3^ Department of Geography Ludwig‐Maximilians‐University of Munich Munich Bavaria Germany; ^4^ Institute for Hygiene and Public Health University Hospital Bonn Bonn North‐Rhine Westphalia Germany; ^5^ Institute of Nutrition and Food Sciences (IEL) Nutritional Epidemiology University of Bonn Bonn North‐Rhine Westphalia Germany

**Keywords:** COVID‐19, eating habits, lifestyle, metropolitan regions, physical activity

## Abstract

COVID‐19 caused profound societal changes to cope rapidly with the new circumstances. The food market changed its quantity, quality, form, and frequency dynamics. Consequently, food‐eating habits and lifestyles like physical exercise likely experienced changes. An online‐based survey was conducted between June 2020 and January 2021 in the metropolitan regions of Rhine Ruhr Metropolis (RRM), Greater São Paulo (GSP), other metropolitan regions in São Paulo state (oMRSP), other Brazilian metropolitan regions (oBRMR), and the remaining urban areas in both countries (oUA), representing different contexts of Brazil and Germany. We assessed self‐reported changes in physical activity level, diet quality, self‐reported eating habits, and buying groceries during the first year of the pandemic. In Germany, indoor and outdoor activities increased for 34% of the respondents, while in Brazil, there was a decrease in physical activity for 50% of the participants. The Healthy Eating Index (HEI) scored higher among Brazilians (16.8) than Germans (15.2) on a 0–29 HEI scale. Increased awareness of healthy and sustainable eating habits was observed in GSP (0.7), oMRSP (0.63), oBRMR (0.7), and oUA (0.68) on a scale from no change (0) to change (1). In RRM, an increase in convenience foods was noticed (0.86). Participants reported discomfort with food purchasing due to hygiene measures and avoided going to the supermarket (0.7 on average in Brazil and 0.58 for females using the same 0–1 scale). Also, food supply at the grocery shops was reported to be often unavailable and in lower quantities. A real‐time assessment of self‐reported changes in eating habits and lifestyle during the lockdown in 2020 in different contexts is insightful for rethinking strategies to improve conditions in the post‐COVID‐19 era and prepare for future pandemics.

## Graphical abstract

An online‐based survey was conducted between June 2020 and January 2021 in the metropolitan regions, representing different contexts in Brazil and Germany. We assessed self‐reported changes in 2020 in physical activity level, diet quality, self‐reported eating habits, and buying groceries. A real‐time assessment of self‐reported changes during the lockdown in 2020 in different contexts is insightful for rethinking strategies to improve conditions in the post‐COVID‐19 era and prepare for future pandemics.

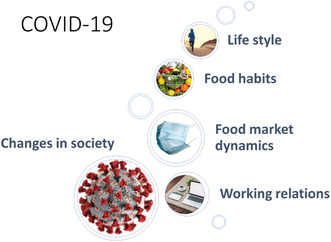

## 1 INTRODUCTION

COVID‐19 conveyed uncertainties and changes in behavior to adapt quickly to disruptions in the food systems, affecting daily life in Brazil and Germany, like in many other middle‐income and OECD countries (Benton, 2020). These profound societal changes transformed behavior, social interactions, eating, and lifestyle habits. Several food system disruptions occurred, changing the dynamic of food markets and increasing the vulnerability of food supply chains (Benton, 2020; Oliveira et al., 2020; Power et al., 2020). In the early stages of the COVID‐19 pandemic, supermarket shelves appeared to be emptied of crucial food and non‐food items in OECD countries (Hobbs, 2020). The lack of labor in food production destabilized the food supply in Germany due to frequent outbreaks and migration restrictions (Mitaritonna & Ragot, 2020; Schneider & Götte, 2022). In addition, the necessary hygiene measures led to uncomfortable shopping experiences due to the regular mandatory use of masks, distance measures, sanitizing fluids, and disinfection of food products, such as observed in the Brazilian strict protocols (Finger et al., 2021; Rizou et al., 2020).

Purchases of pantry items increased worldwide, i.e., frozen and packaged foods, while fresh produce items decreased (IFIC, 2020). Unhealthy eating habits have been associated with emotional eating to divert attention from stress, boredom, and fear caused by the uncertainties of the pandemic (Araiza & Lobel, 2018; Jia et al., 2021; Moynihan et al., 2015). Consumers piled up stocks of food items, increasing the demand for online delivery services (Bakalis et al., 2020). Studies noted a higher volume of ultra‐processed and high‐energy‐density foods purchased in the United States (US), Europe, and Latin America (Morales et al., 2023; Ruíz‐Roso et al., 2020; Sobba et al., 2021). The São Paulo metropolitan area and other middle‐income metropolitan regions reported changes in lifestyle and dietary patterns by increasing high‐calorie foods and reducing healthy food consumption (Jia et al., 2021; Rundle et al., 2020; Sidor & Rzymski, 2020).

Moreover, confinement measures significantly decreased physical activity levels and increased sedentary behavior worldwide (Ammar et al., 2020; García‐Álvarez et al., 2020; Gualano, 2020; Peçanha et al., 2020). The habit of watching television and using the Internet was intensified among adults during the pandemic in the US (Bhutani & Cooper, 2020; Nielsen, 2020). In Spain, alcohol consumption and tobacco use were amplified during quarantine (García‐Álvarez et al., 2020). However, in Italy, a greater adherence to the Mediterranean diet, an increase in physical activity, and a decrease in habits harmful to health, such as smoking, were recorded (Di Renzo et al., 2020).

The literature on the effects of COVID‐19 on eating habits and lifestyle is a growing field, with examples and contexts varying from specific groups within and between countries and regions (Di Renzo et al., 2020; Galali, 2021; Lamy et al., 2022; Thakur & Mathur, 2023). As a middle‐income country, Brazil has particular socioeconomic vulnerabilities, which affected the population differently during the pandemic (Rocha et al., 2021). A study conducted in a southern city in Brazil showed that these specific socioeconomic inequalities determine behavioral and psychological factors of quality of life, including health status and food insecurity (Dumith et al., 2022). Germany, an OECD country, was also impacted by COVID‐19 in the sense that socioeconomic characteristics such as income inequalities, living conditions, and employment status have pushed the vulnerable population at risk of poverty and increased the health burden and food insecurity (Nakazato, 2021; Statistisches Bundesamt, 2021; Wissenschaftlicher Beirat für Agrarpolitik, 2023).

Our study adds the perspective of the impacts of COVID‐19 on the population in metropolitan areas in Brazil (a middle‐income country) and Germany (an OECD country). Cross‐regional empirical research provides valuable insights into the unique challenges and opportunities faced by different regions across the globe when tackling similar issues (Köllner et al., 2018). These urban regions offer different sociocultural contexts, including socioeconomic inequalities, to allow analyses of self‐reported changes in eating habits and lifestyle during the first year of the pandemic, where control measures (e.g., lockdowns) were more prominent because larger cities were typical hotspots of new COVID‐19 infections (Sharifi & Khavarian‐Garmsir, 2020). We intend to investigate the self‐reported changes in urban populations in one country from the global north and one from the global south, showing the same socioeconomic heterogeneity. It is relevant to consider self‐reported behavioral changes to understand the effects on health and well‐being in different geographical locations and use them as lessons learned for future pandemics.

## 2 MATERIAL AND METHODS

### 2.1 Study design, setting, and participants

This cross‐sectional, survey‐based study evaluates self‐reported dietary habits and lifestyle changes during the first year of the COVID‐19 pandemic in metropolitan regions in two different contexts (Brazil and Germany). The survey contains questions about (a) sociodemographic and general information, (b) self‐reported changes in diet behavior and buying groceries, (c) self‐reported changes in physical activity, and (d) a food frequency questionnaire (FFQ). This survey was jointly developed by the University of Bonn and the University of São Paulo by experts in health and nutrition, ecology, and geography. The original German questionnaire was modified to adapt to the Brazilian socio‐cultural context. These modifications relate to demographic and specific questions on alcohol consumption and food consumption. Before data collection, a prior test was conducted with researcher colleagues within the university departments. The survey was available online from June 2020 to January 2021. The recruitment was initiated in Germany on June 14th, 2020, and ended on January 14th, 2021. In Brazil, it was initiated on September 16th, 2020, and ended on January 17th, 2021. It was disseminated via social media and e‐mails to facilitate online participation during the lockdown. Due to the low response rate in Germany, advertisements containing a QR code were distributed in commercial and residential buildings under limited lockdown conditions.

A free and written informed consent form was provided digitally before participation. Only adults who declared themselves to be 18 years old or older were recruited. Participants younger than 18 were directly sent to the end of the survey webpage and excluded. The research was approved by the Research Ethics Committee of the Faculty of Pharmaceutical Sciences, University of São Paulo (CAAE 31781720.9.0000.0067) and the Research Ethics Committee, Center for Development Research (ZEF), University of Bonn, Germany (13c_19), respecting the Declaration of Helsinki (Doenges & Dik, 1964).

We used Cochran's sample size equation with a 95% confidence interval and a 5% margin of error. The percentage of the adult population (18–75 years old) was 69.1% in Brazil and 71.1% in the state of North‐Rhine Westphalia in Germany (IBGE, 2018; IT.NRW, 2023). Thus, a sample size of 329 in Brazil and 316 in Germany was calculated. One thousand twenty‐four participants registered for the survey (*N* = 1024). Although the sample size of the German population is smaller than estimated, the sample has a 6.69% margin of error within a confidence interval of 95%.

### 2.2 Measurements and procedures

#### 2.2.1 Sociodemographic and general information

Information on gender, age, marital status, level of education, number of inhabitants in the household, income at the household level, and ethnicity (nationality in Germany and ethnic groups in Brazil) was collected following the demographic standards of both countries (Bundesamt & Standards, 2016; IBGE, 2008, 2010; IT.NRW, 2013). These sociodemographic variables are dietary and lifestyle behavior determinants and possible confounders (Krieger et al., 2018). To harmonize monthly household income from both countries, we used the Purchasing Power Parity (PPP) conversion factor from the World Bank Database based on the gross domestic product (GDP) and expenditures in international PPP dollars (Int$) (World Bank Group, 2023). The location (city of residency) was used to classify the metropolitan regions based on population density (EUROSTAT, 2021; GV‐ISys, 2019; IBGE, 2021a, 2021b, 2021c, 2021d). Additionally, we collected information on the employment situation before and during COVID‐19 (in the lockdown of 2020) to capture abrupt changes in the working environment and employment status.

#### 2.2.2 Physical activity and general health status

Physical activity assessment followed the validated methods described by the *International Physical Activity Questionnaire* (IPAQ) short form (Craig et al., 2003; van Poppel et al., 2010). This method estimates individual energy expenditures in Metabolic Equivalents of Tasks (METs) expressed in METs minutes/week. The calculation of MET values was obtained by multiplying the time spent (in minutes) on walking (for at least 10 min), moderate (e.g., carrying light weights, cycling at average speed, or swimming at normal speed), or vigorous (e.g., aerobics, running, fast cycling, or fast swimming) activities by the number of days spent on each activity per week. A multiplying factor is added to each activity: walking 3.3, moderate 4.0, and vigorous 8.0. The total physical activity consisted of walking, moderate, and vigorous MET scores. For sitting, it was considered the total number of minutes per day spent sitting. Data processing, cleaning, and normalization procedures were performed using the IPAQ methods (IPAQ, 2005).

Moreover, information on self‐reported changes in physical activities during the COVID‐19 pandemic was collected, considering the participant's perception of being more or less physically active (indoors or outdoors) and the increase in sitting due, among others, to working from home (‘home office’). Information was collected on self‐reported physical health evaluation using five scales (1–5) (“not healthy at all”, “not quite health”, “moderately healthy”, “very healthy”, and “extremely healthy”), self‐reported non‐communicable diseases, smoking habits, pregnancy, or breastfeeding to capture the general health status of the participants.

#### 2.2.3 Diet quality score

Food consumption data were collected using two FFQs. In Brazil, a simplified FFQ was developed with the general survey and included food groups and items based on the Food Guide for the Brazilian population (Brasil, 2014). In Germany, participants completed the EPIC II (Nöthlings et al., 2007), a validated FFQ specifically developed for the German population (Harttig, 2021). We evaluated the dietary quality of both countries using a diet quality indicator, the *Healthy Eating Index* (HEI), adapted from the Mediterranean Diet Score (MDS) (Gil et al., 2015; Trichopoulou et al., 1995; Waijers et al., 2007). HEI sets scores for all food groups common in both countries, using food groups as the main component for scoring. Food frequencies of both FFQ were harmonized (never = 0; every month or less = 0.0164; 2–3 times per month 0.0822; 1–3 times per week = 0.2849; 4–6 times per week = 0.7123; and every day or more = 1.8333) and aggregated to each food group. Each food category's corresponding frequency median values were set as cut‐off criteria, scoring from 0 to 1, with a maximum score of 29 (see Table S1 in the Data S4 for more details).

#### 2.2.4 Self‐reported changes in eating habits and buying groceries

Participants' self‐reported changes in eating habits and buying groceries during COVID‐19 were collected using Likert scale matrices ranging from “to a great extent”, “somewhat”, “very little”, to “not at all”. Options “I do not know/I prefer not to respond” or “not applicable” were also available. These questions were elaborated upon following the questionnaire structure of the iCare study (Bacon et al., 2021). The objective was to collect information on personal perceptions toward eating habits and food supply changes during the lockdown concerning diet quality, consumption of convenience foods, staples, and fresh produce, reasons for dietary changes, including health and sustainability, food purchasing habits, hygiene, shortages in quality or quantity, and financial reasons.

### 2.3 Statistical analysis

General descriptive statistics were applied to sociodemographic and other general information data. We conducted power calculations to compute the sample size, power, and minimum effect size. Robust linear regression analysis evaluated significant differences and correlations between the metropolitan areas and gender in each dependent variable of demographics, PAL, food intake, and HEI. In the specific case of NCDs, each disease was considered a different binary (yes/no) dependent variable in the regression. Logistic regression analysis and the marginal effects were calculated to assess the probability of changes in eating habits and food supply and identify differences between the correlation of sex and metropolitan regions. The Likert scale from “not at all” to “in great extent” was transformed into binary variables (dummies) varying from 0 = no change to 1 = change. STATA vs. 16 (Stata Corp LLC, 2019) was used for all statistical analyses. Data cleaning, categorization, harmonization, and standardization were conducted in SPSS 28 (IBM Corp, 2021).

## 3 RESULTS

### 3.1 Participants

**FIGURE 1:**
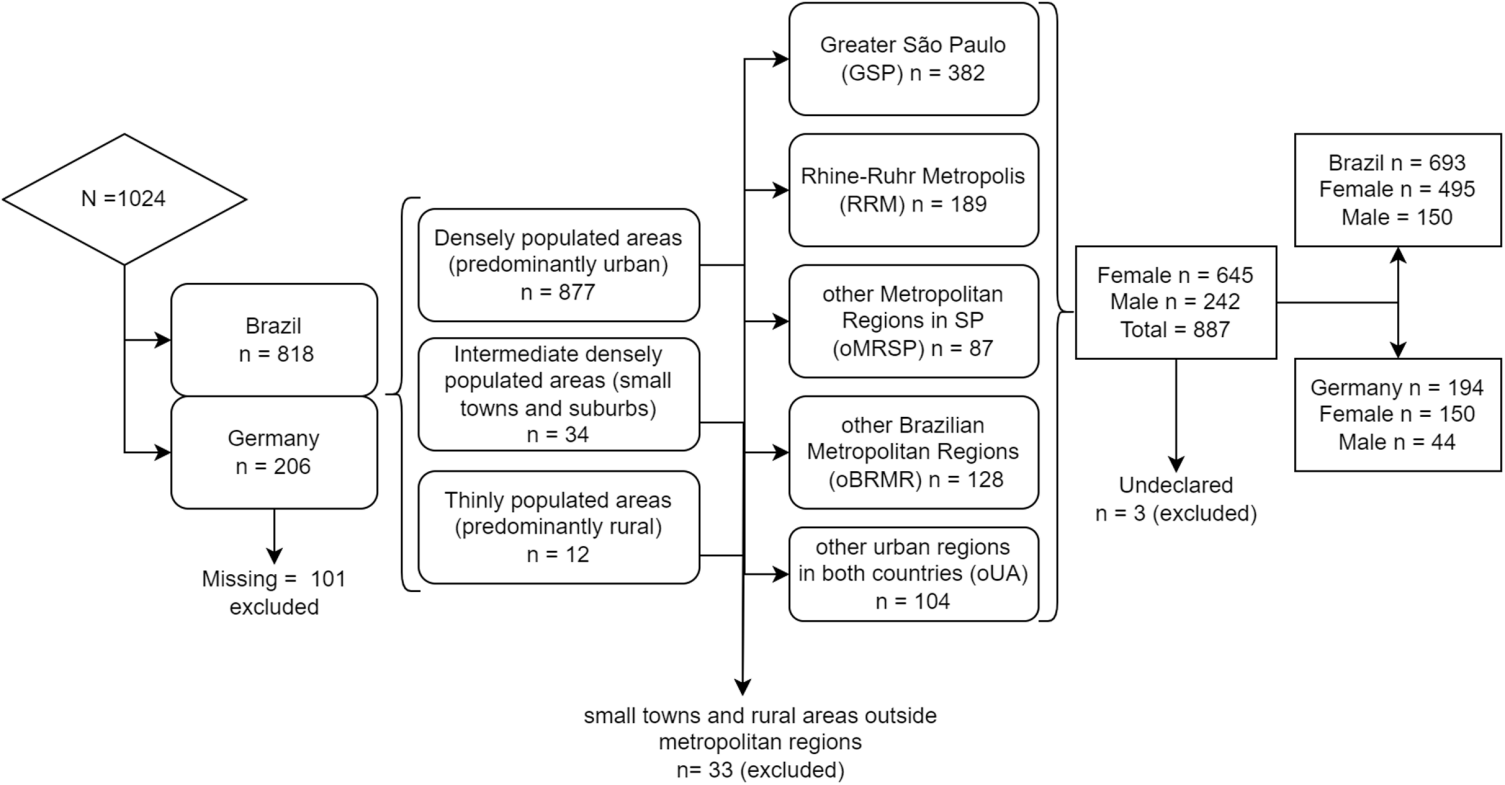
Study participant inclusion and exclusion criteria.

The total number of participants summed up to *n* = 818 in Brazil and *n* = 206 in Germany. Around *n* = 923 (90.1%) responses were considered valid. Missing cases and invalid or incomplete answers were treated as missing (*n* = 101, 9.9%). Power calculation results indicate that no significant effect (<0.80) can be inferred from intermediately populated areas (towns and suburbs) and thinly populated rural areas. Most of the population lived predominantly in urban areas and was located in metropolitan regions (*n* = 890). We included in the analysis all densely populated areas, primarily urban areas and small towns within the metropolitan regions. However, we excluded lesser populated areas not located within any metropolitan region (*n* = 33). The female/male ratio could have been more optimal; however, we decided to include both sexes in the analysis to be consistent, although no significant effect size was observed for Germany's male population (*n* = 44). Undeclared gender was also excluded from the analysis, resulting in a final population size of *N* = 887 participants. The flow chart in Figure 1 illustrates the *n* of participants in each part of the study.

There were 887 valid participants in the study: 693 in Brazil and 194 in Germany. Brazilian participants were from three different metropolitan regions: Greater São Paulo (GSP, *n* = 382), other metropolitan regions in São Paulo State (oMRSP, *n* = 87), and other Brazilian metropolitan regions (oBRMR, *n* = 128). In Germany, the participants were from the Rhine‐Ruhr Metropolis (RRM, *n* = 189). Other urban regions (oUA, *n* = 104) were composed of cities from Brazil (*n* = 98) and Germany (*n* = 6), agglomerated to form a sample with effect.

### 3.2 Characteristics of study participants

The demographic characteristics of the study population are relatively homogenous among different locations: majority female, in the mid‐40's, highly educated, belonging to middle‐upper classes, according to income, ethnicity, and household composition (Table 1). Female participants were predominant (74.4% in Brazil and 73.5% in Germany), with an age average of 44.5 ± 0.5 years old. Most participants declared to be married (47.8%) or single (31.8%). Few participants reported being single but living with a partner (8.4%), divorced or living apart (9.8%), or widowed (1.6%). Regarding ethnicity, most participants in Germany declared that they possess German nationality (87.1% of the 194 valid answers). Only 5.7% had German and other nationalities, 3.1% had different nationalities, and 2.6% had European nationalities. In Brazil, answers regarding participants' self‐declared ethnicity showed a majority of Whites (78.7%), followed by “Pardos” (“brown” in Portuguese for European, and African descendants (Britannica, 2009)) (13%), and a few Asian descendants (3.8%), African descendants (3.3%), and Indigenous (0.1%).

**TABLE 1:** Sociodemographic and health characteristics of selected study participants by metropolitan regions expressed in percentages and mean values, accompanied by standard errors of the mean.

Variables	Categories	Greater São Paulo	Metropolitan regions in São Paulo state	Other Brazilian metropolitan regions	Rhine‐Ruhr Metropolis	Other urban areas	Total
Gender Valid = 887	Female	270 (70.9%)	66 (75.9%)	95 (74.2%)	145 (77.1%)	69 (67.0%)	645 (72.7%)
	Male	111 (29.1%)	21 (24.1%)	33 (25.8%)	43 (22.9%)	34 (33.0%)	242 (27.3%)
Age Valid = 879 Missing = 8	Mean ± Std. Err.	43.9 ± 0.8	42.4 ± 1.4	48.8 ± 1.3***	43.6 ± 1.1	44.8 ± 1.3	44.5 ± 0.5
Marital Status Valid = 887	Married	183 (48.0%)	47 (54.0%)	57 (44.5%)	85 (45.2%)	52 (50.5%)	424 (47.8%)
	Single	127 (33.3%)	23 (26.4%)	44 (34.4%)	53 (28.2%)	35 (34.0%)	282 (31.8%)
	Single but living with a partner / registered civil partnership	20 (5.3%)	5 (5.8%)	5 (3.9%)	38 (20.2%)	6 (5.8%)	74 (8.4%)
	Divorced/living apart	40 (10.5%)	9 (10.3%)	19 (14.8%)	11 (5.9%)	8 (7.8%)	87 (9.8%)
	Widowed	8 (2.1%)	2 (2.3%)	2 (1.6%)	0 (0.0%)	2 (1.9%)	14 (1.6%)
	Undeclared	3 (0.8%)	1 (1.2%)	1 (0.8%)	1 (0.5%)	0 (0.0%)	6 (0,7%)
Nationality (Germany) Valid = 194	German nationality	‐	‐	‐	164 (87.2%)	5 (83.3%)	169 (87.1%)
	German with other nationalities	‐	‐	‐	10 (5.3%)	1 (16.7%)	11 (5.7%)
	European nationality	‐	‐	‐	5 (2.7%)	0 (0.0%)	5 (2.6%)
	Other nationality	‐	‐	‐	6 (3.2%)	0 (0.0%)	6 (3.1%)
	Undeclared				3 (1.6%)	0 (0.0%)	3 (1.5%)
Ethnicity Valid = 690 Missing = 3	Whites	314 (82.6%)	66 (75.9%)	99 (77.9%)	‐	64 (66.7%)	543 (78.7%)
	African descendants	9 (2.4%)	2 (2.3%)	7 (5.5%)	‐	5 (5.2%)	23 (3.3%)
	“Pardos”^a^	32 (8.4%)	16 (18.4%)*	17 (13.4%)	‐	25 (26.0%)***	90 (13.0%)
	Asian descendants	21 (5.5%)	3 (3.4%)	0 (0.0%)	‐	2 (2.1%)	26 (3.8%)
	Indigenous	0 (0.0%)	0 (0.0%)	1 (0.8%)	‐	0 (0.0%)	1 (0.1%)
	Undeclared	4 (1.1%)	0 (0.0%)	3 (2.4%)	3 (1.6%)	0 (0.0%)	7 (1.0%)
Education Valid = 887	Primary education (incomplete)	0 (0.0%)	1 (1.1%)	0 (0.0%)	1 (0.5%)	1 (1.0%)	3 (0,3%)
	Primary education or equivalent	2 (0.5%)	3 (3.5%)	1 (0.8%)	0 (0.0%)	2 (1.9%)	8 (0.9%)
	Secondary education (incomplete)	2 (0.5%)	1 (1.1%)	1 (0.8%)	0 (0.0%)	1 (1.0%)	5 (0.6%)
	Secondary education or equivalent	13 (3.4%)	8 (9.2%)	2 (1.5%)	12 (6.4%)	10 (9.7%)	45 (5.1%)
	General Higher Education Entrance Qualification “Abitur”	‐	‐	‐	55 (29.3%)	2 (1.9%)	57 (6.4%)
	Technical post‐secondary higher education	11 (2.9%)	4 (4.6%)	1 (0.8%)	0 (0.0%)	3 (2.9%)	19 (2.1%)
	Bachelor or equivalent (incomplete)	63 (16.5%)	10 (11.5%)	11 (8.6%)	1 (0.5%)	16 (15.5%)	101 (11.4%)
	Advanced technical college	‐	‐	‐	5 (2.7%)	0 (0.0%)	5 (0.6%)
	University level (Bachelor/Master/Doctor)	289 (75.9%)	60 (69.0%)	111 (86.7%)	113 (60.1%)	68 (66.0%)	641 (72.3%)
	Preferred not to respond	1 (0.3%)	0 (0.0%)	1 (0.8%)	1 (0.5%)	0 (0.0%)	3 (0.3%)
Household size valid = 887	Mean ± Std. Err.	2.7 ± 0.1	2.9 ± 0.1	2.5 ± 0.1	2.4 ± 0.1*	2.9 ± 0.1	2.6 ± 0.0
Household monthly income (PPP Int$^b^) Valid = 811 Missing = 76	Mean ± Std. Err.	4164.02 ± 118.00	3142.17 ± 226.32***	4441.37 ± 159.03	5904.21 ± 369.92***	4118.56 ± 269.42	4472.66 ± 108.37
Self‐reported physical health evaluation Valid = 884, Missing = 3	Extremely healthy	21 (5.5%)	4 (4.6%)	10 (7.9%)	25 (13.4%)	8 (7.8%)	68 (7.7%)
	Very healthy	121 (31.9%)	30 (34.5%)	57 (44.9%)	90 (48.1)	32 (31.1%)	330 (37.3%)
	Moderately healthy	177 (46.6%)	37 (42.5%)	51 (40.1%)	65 (34.8%)***	44 (42.7%)	374 (42.3%)
	Not quite healthy	48 (12.6%)	12 (13.8%)	9 (7.1%)	7 (3.7%)***	14 (13.6%)	90 (10.2%)
	Not healthy at all	13 (3.4%)	4 (4.6%)	0 (0.0%)	0 (0.0%)	5 (4.8%)	22 (2.5%)
Self‐reported non‐communicable diseases (NCDs) Valid = 884, Missing = 3	No chronic illnesses	166 (38.8%)	42 (38.9%)	68 (40.7%)	75 (42.6%)	42 (38.9%)	393 (39.8%)
	Cardiovascular diseases	22 (5.1%)	6 (5.6%)	10 (6.0%)	4 (2.3%)	11 (10.2%)	53 (5.4%)
	Cancer	7 (1.6%)	1 (0.9%)	0 (0.0%)	3 (1.7%)	1 (0.9%)	12 (1.2%)
	Nutrition deficiencies	7 (1.6%)	3 (2.8%)	2 (1.2%)	1 (0.6%)	2 (1.9%)	15 (1.5%)
	Diabetes	28 (6.5%)	4 (3.7%)	3 (1.8%)	3 (1.7%)**	3 (2.8%)	41 (4.2%)
	Dyslipidaemia	38 (8.9%)	10 (9.3%)	13 (7.8%)	0 (0.0%)	8 (7.4%)	69 (7.0%)
	Thyroid problems	43 (10.0%)	14 (13.0%)	22 (13.2%)	9 (5.1%)*	3 (2.8%)*	91 (9.2%)
	Food intolerance	12 (2.8%)	4 (3.7%)	11 (6.6%)*	20 (11.4%)***	4 (3.7%)	51 (5.2%)
	Hypertension	48 (11.2%)	12 (11.1%)	20 (12.0%)	19 (10.8%)	16 (14.8%)	115 (11.7%)
	Allergies	9 (2.1%)	1 (0.9%)	0 (0.0%)	7 (4.0%)	1 (0.9%)	18 (1.8%)
	Arthritis	2 (0.5%)	0 (0.0%)	2 (1.2%)	0 (0.0%)	0 (0.0%)	4 (0.4%)
	Asthma	13 (3.0%)	1 (0.9%)	3 (1.8%)	4 (2.3%)	2 (1.9%)	23 (2.3%)
	Autoimmune diseases	1 (0.2%)	1 (0.9%)	2 (1.2%)	2 (1.1%)	1 (0.9%)	7 (0.7%)
	Chronic lung diseases	1 (0.2%)	0 (0.0%)	1 (0.6%)	1 (0.6%)	0 (0.0%)	3 (0.3%)
	Colitis	0 (0.0%)	0 (0.0%)	0 (0.0%)	6 (3.4%)	0 (0.0%)	6 (0.6%)
	Endometriosis	0 (0.0%)	1 (0.9%)	0 (0.0%)	2 (1.1%)	1 (0.9%)	4 (0.4%)
	Fibromyalgia	1 (0.2%)	0 (0.0%)	2 (1.2%)	3 (1.7%)	1 (0.9%)	7 (0.7%)
	Digestive disorders	3 (0.7%)	1 (0.9%)	0 (0.0%)	0 (0.0%)	1 (0.9%)	5 (0.5%)
	Kidney diseases	1 (0.2%)	1 (0.9%)	1 (0.6%)	1 (0.6%)	0 (0.0%)	4 (0.4%)
	Mental health issues	6 (1.4%)	1 (0.9%)	1 (0.6%)	1 (0.6%)	4 (3.7%)	13 (1.3%)
	Migraine	4 (0.9%)	1 (0.9%)	0 (0.0%)	3 (1.7%)	1 (0.9%)	9 (0.9%)
	Multiple sclerosis	1 (0.2%)	1 (0.9%)	0 (0.0%)	2 (1.1%)	0 (0.0%)	4 (0.4%)
	Musculoskeletal disorder	3 (0.7%)	1 (0.9%)	2 (1.2%)	3 (1.7%)	0 (0.0%)	9 (0.9%)
	Osteoarthritis	2 (0.5%)	0 (0.0%)	1 (0.6%)	5 (2.8%)*	3 (2.8%)*	11 (1.1%)
	Tendonitis	2 (0.5%)	0 (0.0%)	1 (0.6%)	0 (0.0%)	0 (0.0%)	3 (0.3%)
	Neurodermatitis	1 (0.2%)	0 (0.0%)	0 (0.0%)	0 (0.0%)	0 (0.0%)	1 (0.1%)
	Obesity	1 (0.2%)	0 (0.0%)	1 (0.6%)	1 (0.6%)	0 (0.0%)	3 (0.3%)
	Other diseases	6 (1.4%)	2 (1.9%)	1 (0.6%)	1 (0.6%)	3 (2.8%)	13 (1.3%)
Pregnant/Nursing Valid = 884, missing = 3	No	281 (73.9%)	62 (71.3%)	96 (75.6%)	179 (95.7%)	75 (72.8%)	693 (78.4%)
	Pregnant	2 (0.5%)	2 (2.3%)	1 (0.8%)	2 (1.1%)	1 (1.0%)	8 (0.9%)
	Nursing	9 (2.4%)	3 (3.4%)	2 (1.6%)	2 (1.1%)	2 (1.9%)	18 (2.0%)
	Not applicable	88 (23.4%)	20 (23.0%)	28 (22.0%)	4 (2.1%)	25 (24.3%)	165 (18.7%)
Smoking Valid = 882 Missing = 5	Non‐smoker	321 (84.7%)	73 (83.9%)	117 (92.1%)	171 (91.4%)	92 (90.2%)	774 (87.8%)
	Smoker	58 (15.3%)	14 (16.1%)	10 (7.9%)	15 (8.0%)	10 (9.8%)	107 (12.1%)
	Prefer not to respond	0 (0.0%)	0 (0.0%)	0 (0.0%)	1 (0.6%)	0 (0.0%)	1 (0.1%)

*Note*: Selected participants, *N* = 887 (See Figure 1). The values displayed are within a 95% confidence interval. Significance: *p*‐values .05*, .01**, and .001*** based on robust linear regression.

^a^ ^“^Brown” in Portuguese for European, and African descendants (Britannica, 2009).

^b^ International Purchasing Power Parity dollars.

As for education, participants showed a higher educational level: 72.3% obtained a university‐level degree, 11.4% were initiated at the university level, but it was incomplete by the time of the study, 6.4% had the general higher education entrance qualification (“Abitur” for Germany), and very few participants had below secondary education level. The mean household size was 2.6. Therefore, the participants lived together with one or two others. The average household monthly income of the whole sample was Int$ 4472.66 ± 108.37 6, significantly higher in the RRM (5904.21 ± 369.92, *p* ≤ .001), representing middle‐upper social classes.

The results on the general health status showed that most participants self‐declared to be healthy: “Moderately healthy” (42.3%), “very healthy” (37.3%), and “extremely healthy” (7.7%). The percentage of participants who self‐declared to be “not quite healthy” was 10.2%, particularly within the metropolitan locations in Brazil (*p* ≤ .001). In terms of self‐declared diet‐related non‐communicable diseases (NCDs) – considering a multiple‐choice answer selection – the results from the most reported to the lowest reported were declared as follows: No chronic illnesses (39.8%), hypertension (11.7%), thyroid problems (9.2%), dyslipidaemia (7%), cardiovascular diseases (5.4%), food intolerance (5.2%), diabetes (4.2%), asthma (2.3%), allergies (1.8%), nutritional deficiencies (1.5%), mental health diseases (1.3%), other diseases (1.3%), cancer (1.2%), and osteoarthritis (1.1%). NCDs reported less than 1%, which can be seen in Table 1. Most participants were not pregnant (78.4%) or not applicable (18.7%); only 8 participants (0.9%) reported being pregnant and 18 (2%) were nursing. Most participants were non‐smokers (87.8%); 12.1% said they were smokers.

### 3.3 Employment situation during COVID‐19

**FIGURE 2:**
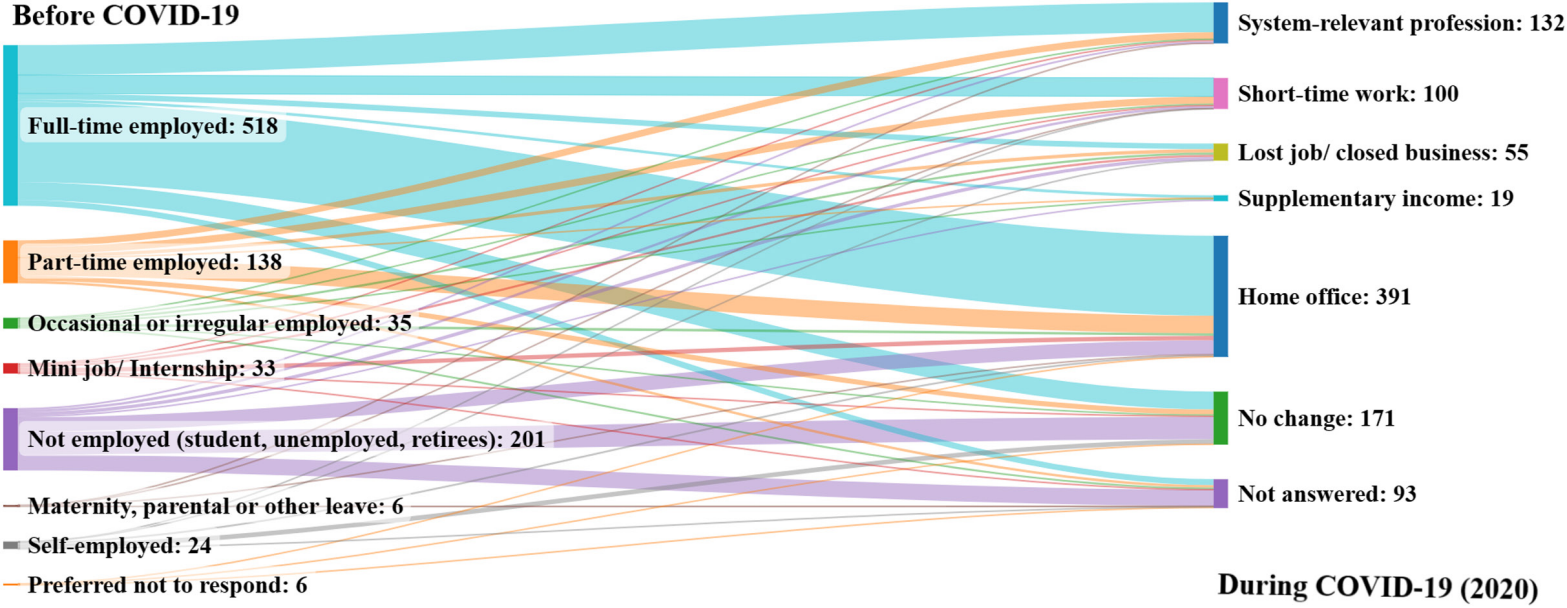
Employment situation before and during the COVID‐19 pandemic in 2020.

The employment situation during COVID‐19 in 2020 changed from very little to no change (82.5%), considering multiple‐choice responses. Figure 2 shows the flows of change that occurred in each type of employment situation. Around 40% of employment changed to home office, 18% reported no change, 14% constituted system‐relevant professions, and 10% shifted to short‐time work. Full‐time employment changed to home office (50%), system‐relevant profession (18%), and short‐time work (11%). Sixty‐two percent of the self‐employed and 36% of the unemployed reported no change during COVID‐19.

### 3.4 Physical activity level (PAL)

**FIGURE 3:**
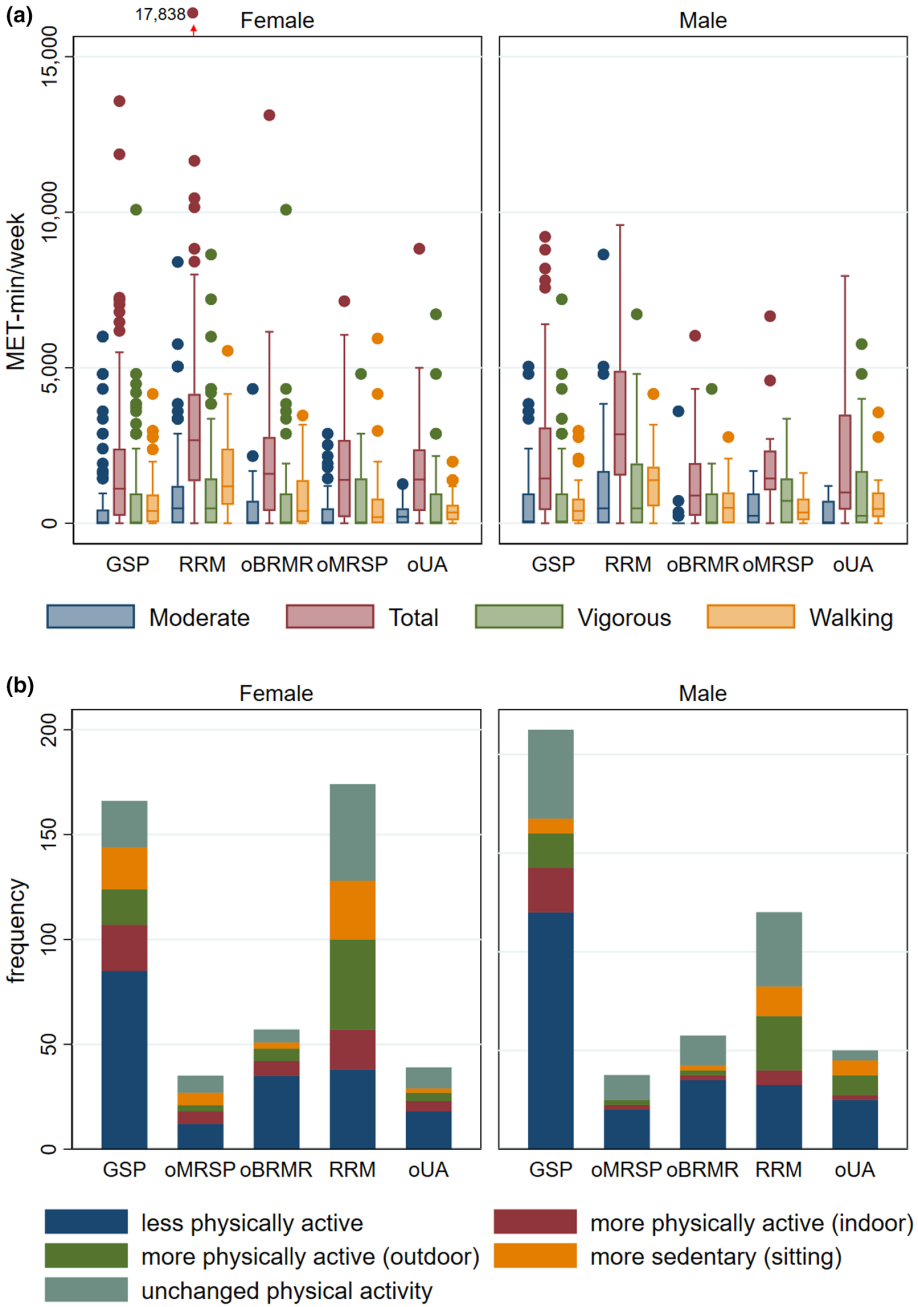
Physical Activity Level (PAL) and self‐reported changes in physical activity during COVID‐19. (a) Metabolic Equivalents of Tasks (MET‐min/week) of vigorous, moderate, and walking activities by sex and metropolitan regions. (b) Self‐reported changes in physical activity during the pandemic in frequency of responses. Boxplots represent Q1: lower quartile, Q2: median, and Q3: upper quartile; error bars are upper and lower adjacent values; and markers are outside values at 95% confidence intervals. Valid answers: *n* = 569, confidence interval at 95%. GSP, Greater São Paulo; oBRMR, other Brazilian metropolitan regions; oMRSP, other metropolitan regions in São Paulo state; oUA, other urban areas; RRM, Rhine‐Ruhr Metropolis.

In general, metropolitan regions in Brazil presented lower levels and a self‐reported reduction of physical activity during the pandemic. In contrast, the activity level in Germany was higher, with an observed increase during COVID‐19. Linear regression results showed a significant difference in the RRM (*p* < .001), which presented higher mean values for both females and males (3282 ± 233.1 and 3484.9 ± 376.8 MET‐min/week, respectively (*p* ≤ .05)) compared to the other metropolitan and urban areas and both sexes (from 1827.9 to 1958.7 MET‐min/week) (Figure 3a). Regarding physical activity level (PAL), walking (*p* = .005) and vigorous (*p* = .042) physical activity levels presented significantly higher means (900.0 ± 64.4 and 832.6 ± 62.3, respectively) compared to moderate levels, with considerably higher means in RRM compared to all other metropolitan regions. These results are complemented by the self‐reported changes in physical activity levels during COVID‐19 (Figure 3b). Nearly 50% (on average) reported being less physically active during the pandemic in the metropolitan regions of GSP, oMRSP, and oBRMR. In contrast, RRM responses indicated a perception of being more physically active outdoors and indoors (34% together), plus having an unchanged high activity level (27%). Around 10% of the participants reported feeling more sedentary due to increased working hours in the home office, with an average of 6.3 ± 0.1 hours per day spent sitting. Additional results are in the Data S2 and S4.

### 3.5 Healthy eating index (HEI)

**FIGURE 4:**
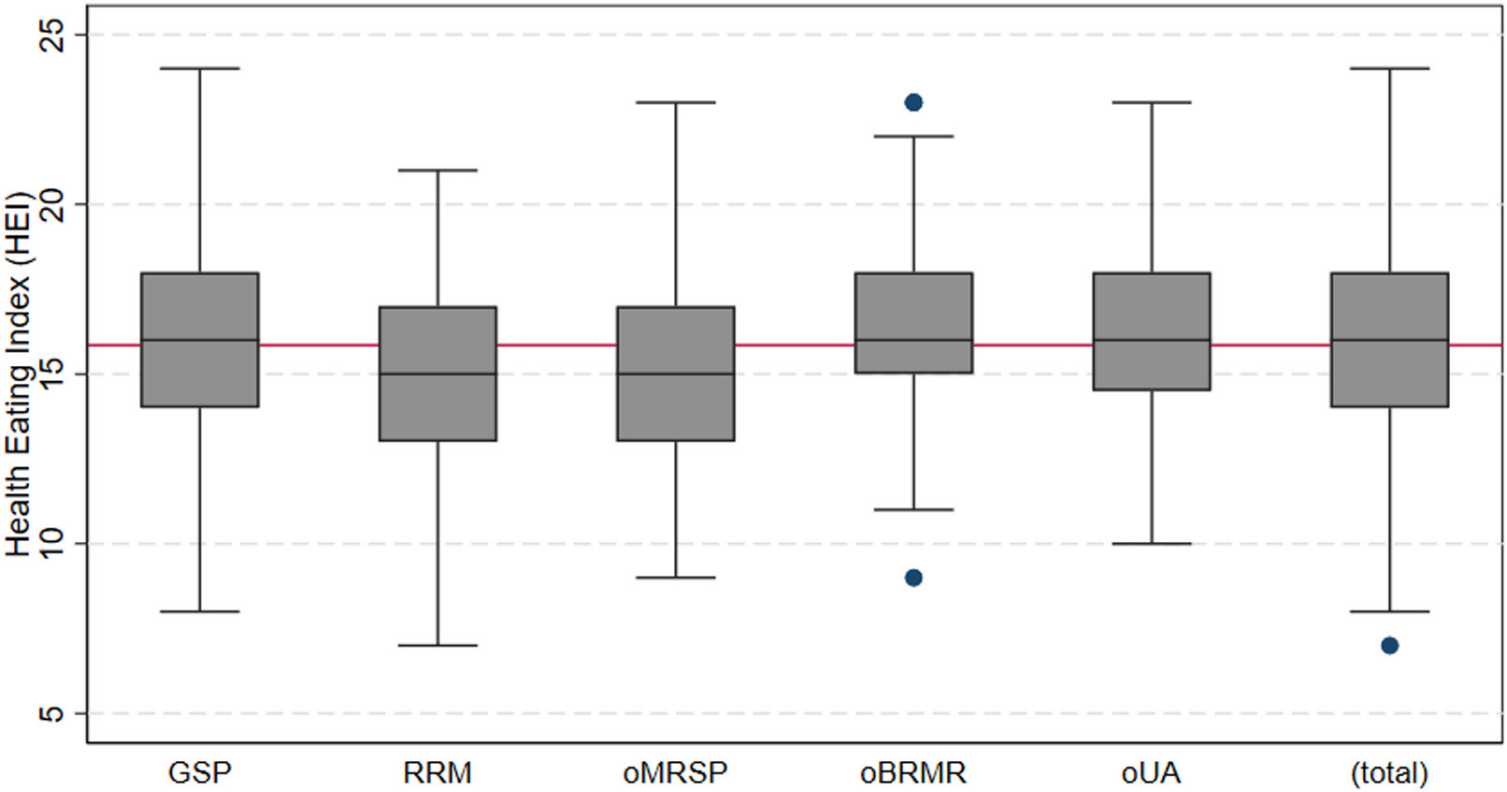
Healthy Eating Index (HEI) by metropolitan regions. Boxplots represent lower quartile (25% percentile), median, and upper quartile (75% percentile); error bars are upper and lower adjacent values; markers are outside values at 95% confidence intervals; and red lines represent total mean values. Scale from 0 to 29. GSP, Greater São Paulo; oMRSP, other metropolitan regions in São Paulo state; oBRMR, other Brazilian metropolitan regions; RRM, Rhine‐Ruhr Metropolis; oUA, other urban areas.

Results from a robust regression analysis demonstrated no significant differences between males and females within the same locations. However, significant differences were observed across metropolitan regions. HEI was significantly higher (*p* ≤ .05) in oBRMR (16.38 ± 0.26), oUA (16.30 ± 0.33), and GSP (15.90 ± 0.16) compared to oMRSP (15.36 ± 0.26) and RRM (15.21 ± 0.24) on a scale from 0 to 29 (Figure 4). HEI results can be interpreted by looking at the differences in food consumption based on the reported food frequencies (Table 2). Although RRM consumed significantly more bread, vegetables, fruits, and nuts, it also had a higher frequency of consumption of cheese, sausages, processed foods, desserts and sweets, coffee, juices (processed), ready‐to‐eat meals, water, butter, and oil than the other regions, with some exceptions. The consumption of beer was higher in oUA, rice and pasta in oMRSP, and cheese in oBRMR. In GSP, oMRP, oBRMR, and oUA, consumption was higher for carbohydrates such as cereals, rice and pasta, pulses, potatoes, natural juices, and proteins such as meat, poultry, and eggs than in the German metropolis. For details, see Data S3.

**TABLE 2:** Means and respective standard errors of harmonized food frequencies of selected participants by food group in each metropolitan region.

Food groups	GreaterSão Paulo		Metropolitan regions in São Paulo state		Other Brazilian metropolitan regions		Rhine‐Ruhr Metropolis		Other urban areas	
	Mean	Std. err.	Mean	Std. err.	Mean	Std. err.	Mean	Std. err.	Mean	Std. err.
Bread	0.65	0.02	1.53**	0.04	0.63	0.04	0.61	0.04	0.78*	0.04
Cereals	0.49	0.02	0.33**	0.03	0.42	0.04	0.47	0.04	0.53	0.04
Rice and pasta	1.04	0.03	0.34**	0.02	1.27*	0.07	1.01	0.06	1.16	0.07
Pulses	0.52	0.02	0.17**	0.01	0.57	0.04	0.54	0.04	0.70**	0.03
Potatoes	0.34	0.02	0.24**	0.02	0.33	0.03	0.37	0.03	0.37	0.03
Vegetables	0.75	0.02	1.60**	0.04	0.73	0.03	0.76	0.03	0.80	0.04
Fruits	0.73	0.02	1.59**	0.04	0.67	0.04	0.75	0.03	0.75	0.05
Nuts	0.31	0.02	0.75**	0.05	0.26	0.04	0.33	0.03	0.26	0.04
Milk	0.44	0.02	0.51	0.05	0.42	0.05	0.42	0.04	0.53	0.04
Yogurt	0.29	0.02	0.34	0.03	0.28	0.04	0.29	0.03	0.22	0.03
Cheese	0.53	0.02	0.96**	0.05	0.46	0.04	0.63*	0.03	0.61	0.04
Meat	0.47	0.02	0.18**	0.02	0.53	0.04	0.46	0.03	0.53	0.03
Poultry	0.44	0.02	0.07**	0.01	0.51	0.03	0.45	0.03	0.49	0.03
Sausages	0.18	0.01	0.37**	0.04	0.20	0.03	0.16	0.02	0.22	0.03
Fish	0.16	0.01	0.17	0.01	0.11**	0.01	0.16	0.02	0.12*	0.02
Eggs	0.47	0.02	0.31**	0.02	0.48	0.04	0.51	0.03	0.55	0.04
Ready‐to‐eat meals	0.17	0.01	0.31**	0.02	0.20	0.02	0.13	0.02	0.18	0.02
Ultra‐processed foods	0.23	0.02	1.15**	0.05	0.20	0.03	0.19	0.02	0.29	0.05
Dessert & sweets	0.25	0.02	0.31**	0.03	0.20	0.03	0.22	0.03	0.23	0.03
Coffee & tea	0.80	0.02	1.68**	0.04	0.80	0.04	0.75	0.03	0.87	0.05
Juice (processed)	0.16	0.02	0.29**	0.04	0.23	0.04	0.09*	0.02	0.13	0.03
Juice (natural)	0.30	0.02	0.07**	0.01	0.33	0.04	0.37	0.03	0.32	0.04
Soft drinks	0.18	0.02	0.26*	0.04	0.19	0.03	0.20	0.03	0.18	0.03
Water	0.95	0.01	1.79**	0.02	0.98	0.01	0.98	0.01	1.04**	0.02
Butter	0.61	0.02	0.70*	0.04	0.49*	0.05	0.51*	0.04	0.65	0.04
Oils	0.69	0.02	1.75**	0.03	0.64	0.05	0.72	0.03	0.78	0.05
Beer	0.15	0.01	0.14	0.02	0.18	0.03	0.12	0.02	0.21*	0.03
Spirits	0.10	0.01	0.04**	0.00	0.06	0.01	0.06	0.02	0.07	0.02
Wine	0.12	0.01	0.21**	0.02	0.06**	0.01	0.14	0.02	0.12	0.02
Mixed drinks	0.04	0.01	0.03	0.01	0.02*	0.01	0.03	0.01	0.02*	0.01

Abbreviations: GSP, Greater São Paulo; oBRMR, other Brazilian metropolitan regions; oMRSP, other metropolitan regions in São Paulo state; oUA, other urban areas; RRM, Rhine‐Ruhr Metropolis.

*Note*: Reference frequency: (never = 0; every month or less = 0.0164; 2–3 times per month 0.0822; 1–3 times per week = 0.2849; 4–6 times per week = 0.7123; every day or more = 1.8333). Significance: *p ≤ .05, **p ≤ .001 robust linear regression.

### 3.6 Self‐reported changes in eating habits and buying groceries

**FIGURE 5:**
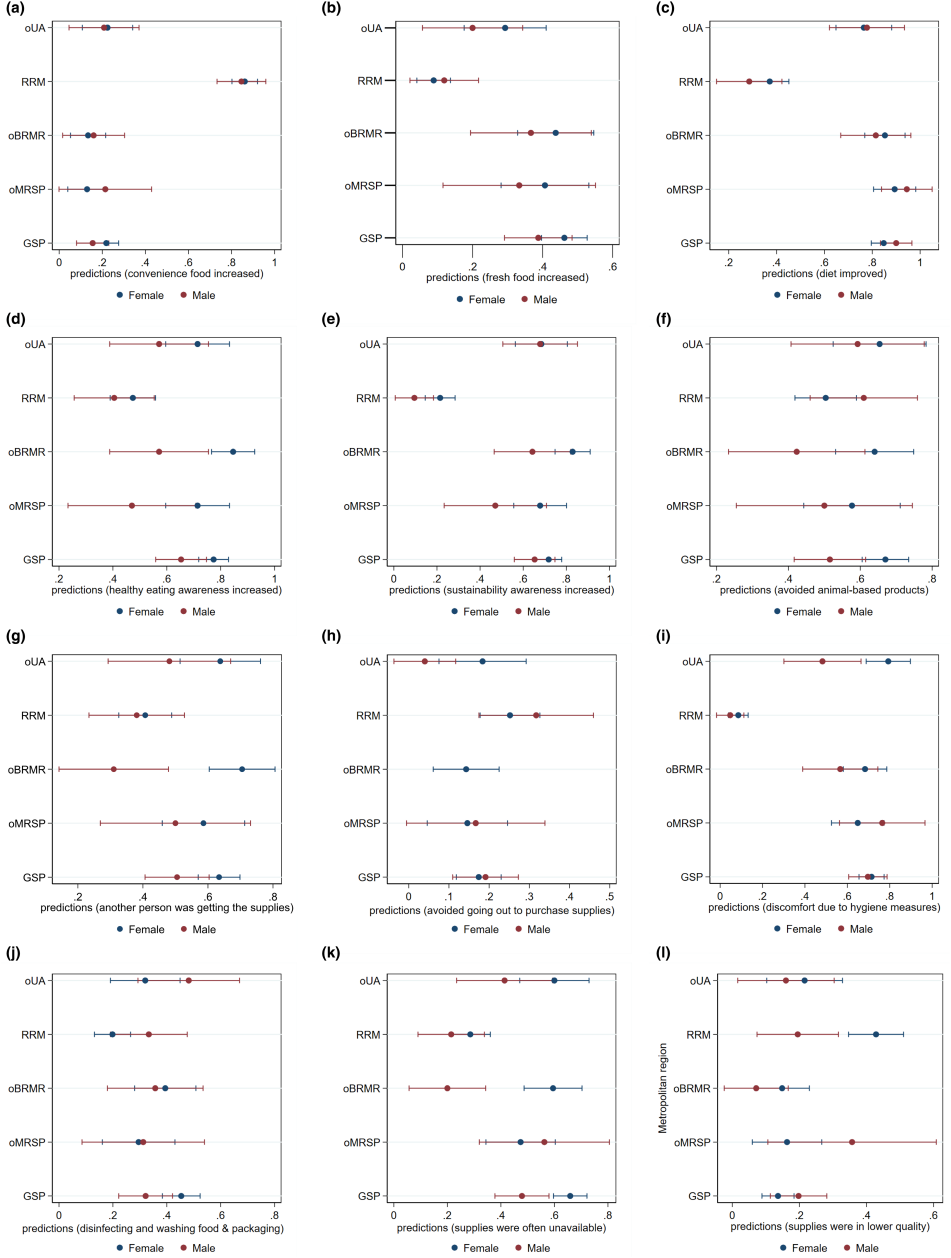
Self‐reported changes in eating habits and buying groceries between sexes and the different metropolitan regions. Dots represent adjusted predictions from no change (0) to change (1) of the logistic regression correlations between sex and metropolitan areas. Error bars represent the 95% confidence intervals. GSP, Great São Paulo; oBRMR, other Brazilian metropolitan regions; oMRSP, other metropolitan regions in São Paulo state; oUA, other urban areas; RRM, Rhine‐Ruhr Metropolis. Only relevant and significant results are shown.

Logistic regression analysis results ranging from self‐reported 0 = no change to 1 = change demonstrated that results differed depending on gender and metropolitan regions (Figure 5). A change related to the increase of fresh food (Figure 5b), improvement of the diet (Figure 5c), enhanced awareness of healthy eating and sustainability (Figure 5d,e), and avoidance of eating animals (Figure 5f) was noticeable in Brazil, while in Germany, there was an increase in the consumption of convenience food (Figure 5a). Regarding changes in buying groceries, participants reported that they avoided going out to the grocery shops (Figure 5h), and food supplies were often of lower quality in Germany (Figure 5l). In Brazil, on the other hand, the changes related to having another person getting the supplies (Figure 5g), discomfort during purchase (Figure 5i), disinfecting food packaging (Figure 5j), and supplies were often observed as unavailable (Figure 5k).

Looking into the different metropolitan regions, the RRM had a higher probability of increasing the consumption of convenience food (0.86 ± 0.02, *p* < .000) compared to other areas (0.14–0.21) (Figure 5a). Also, the perception that the diet improved was higher in GSP (0.86 ± 0.02), oMRSP (0.91 ± 0.03), oBRMR (0.84 ± 0.04), and oUA (0.77 ± 0.05) than in the RRM (0.35 ± 0.03, *p* < .000) (Figure 5c). Enhanced awareness of sustainability was significantly lower in the RRM (0.18 ± 0.03, *p* < .000) than in the other locations (GSP = 0.70 ± 0.03; oMRSP = 0.63 ± 0.06; oBRMR = 0.78 ± 0.04; and oUA = 0.68 ± 0.05) (Figure 5e). Discomfort during food purchasing due to the restricted hygiene measures has a significantly higher level of probability (*p* < .000) in GSP (0.71 ± 0.02), oMRSP (0.67 ± 0.5), oBRMR (0.76 ± 0.02), and oUA (0.68 ± 0.05), compared to RRM (0.08 ± 0.02) (Figure 5i). Disinfecting food packaging had a significantly higher likelihood (*p* = .001) in GSP, oMRSP, oBRMR, and oUA (0.3 ± 0.06 to 0.41 ± 0.03) than in RRM (0.23 ± 0.03) (Figure 5j).

When comparing gender, the probability of increased healthy eating awareness was significantly higher in females (0.69 ± 0.02) than in males (0.56 ± 0.03, *p* < .001) (Figure 5d). Enhanced understanding of sustainability was also significantly higher in females (0.21 ± 0.03) than in males (0.09 ± 0.04, *p* = .037). Moreover, avoiding eating animal products was substantially higher in females (0.61 ± 0.02, *p* = .047) than in males (0.53 ± 0.03) (Figure 5f). Females also had a higher probability of having help from another person to get food supplies (0.58 ± 0.02, *p* = .001) than males (*p* < .000) (Figure 5g). For females, the food supply was significantly more unavailable (0.53 ± 0.02, *p* < .000), especially in oBRMR. In oBRMR, gender differences appeared more pronounced than in other regions in Figure 5g,k. More results are available in the Data (S1 and S4).

## 4 DISCUSSION

Our findings show distinct self‐reported eating habits and lifestyle changes in the Brazilian and German metropolitan regions. Although comparing these two countries is not the study's main objective, discussing these differences within their socio‐cultural contexts is important. While the Brazilian population's concerns were mostly related to improving eating habits and hygiene measures to maintain health, in Germany, priority was given to more physical activity outdoors and indoors to improve human well‐being during the confinement period. Participants suffered changes in the working environment, such as becoming essential workers, transferring to home office work, short‐time work, or even losing their jobs or businesses. The perception of the transmission risk made many participants avoid supermarkets and increase the recommended hygiene measures (i.e., long queues, distancing, use of masks, and disinfectants). Food availability and quality have also created perceptions of food insecurities in the food supply.

Beneficial changes in eating habits were observed in the metropolitan regions of GSP, oMRSP, oBRMR, and oUA, concerning increased consumption of fresh food, awareness of healthy and sustainable eating, and a higher HEI than in the RRM. Traditional Brazilian food consumption is based on whole foods (rice, beans, tubers, and vegetables) (Brasil, 2014). However, in recent years, the Brazilian diet has possibly worsened due to reduced vegetables and fruit and increased consumption of ultra‐processed foods (De Carvalho et al., 2021). Differences in eating habits in our study can be explained by the characteristics of the population, composed of many urban middle‐aged and highly educated women, and by the working‐time flexibility in the home office, which could have influenced the decision to eat healthier and more sustainably. Our survey did not include questions about who was responsible for purchasing and preparing meals. This would provide insights into whether females play an important role in household decision‐making regarding food and nutrition.

It is essential to highlight that COVID‐19 has affected food security in several parts of the world (FAO‐WFP, 2020). The pandemic exposed vulnerabilities in Brazil. The inability to cope with the increasing unemployment and poverty rates provoked around 9 million people to be unable to generally afford a meal in 2020, despite the government's emergency measures (De Carvalho et al., 2021). A recent study in Germany also demonstrated that 3.5% of the population is still at risk of food insecurity even after COVID‐19, especially children and older adults (WBAE & BMEL, 2023). Therefore, our findings must be interpreted with limitations because they represent perceived changes in the middle‐upper‐class urban population and do not consider the vulnerable food‐insecure population amid COVID‐19.

A more significant concern about hygiene and disinfection was observed in GSP, oMRSP, oBRMR, and oUA. Brazilian health authorities highly recommend hygiene measures and personal protection, including everyday food hygiene and disinfecting procedures of food products and packaging, such as washing with water, soap, and alcohol‐based sanitizers (Finger et al., 2021). Moreover, the concern about “cleanliness” is personally valued and fundamentally cultural in Brazil (Barbosa & Veloso, 2014), which might have been extrapolated due to the infection risk and the unequal and insufficient access to health care (Aquino et al., 2020). Another phenomenon during the pandemic is the expansion in food delivery, which explains why many participants have “another person getting the [food] supplies”. The delivery industry in Brazil was intensified in the digital environment via mobile apps to deliver ready‐to‐eat foods and beverages that are convenient to the consumer (Botelho et al., 2020). We should have considered whether employment was formal or informal in our questionnaire. However, informal work, such as delivery services, was particularly affected during the pandemic, lacking labor rights and increasing health risks (Defossez, 2022; Tran et al., 2022).

In RRM, the German metropolis, many participants reported consuming more convenience foods, staples (such as bread), and less fresh produce. Nevertheless, the intake of fruits and vegetables in the RRM was still significantly higher than in other metropolitan regions. This was corroborated by the fact that participants declared reducing the frequency of visiting supermarkets and convenience foods as easier to store. Consuming “comfort food” to satisfy anxiety cravings increased during the pandemic in Italy (Scarmozzino & Visioli, 2020). The RRM metropolis has an average German western‐type diet, with a higher consumption frequency of bread and processed foods, including sausages, dairy, and sweets (Paris et al., 2022, 2023). Additionally, food supplies were often perceived as of lower quality. COVID‐19 made the food safety issues in the food supply chain more evident, including socioeconomic vulnerabilities and health risks (Marchant‐Forde & Boyle, 2020; Rizou et al., 2020). Many disruptions occurred in the food supply (Benton, 2020; Power et al., 2020) caused by less migration of seasonal workers during the harvesting season, increased socioeconomic inequalities, and frequent COVID‐19 outbreaks among workers in the food industry, especially in meat processing facilities (Mitaritonna & Ragot, 2020; Schneider & Götte, 2022).

Another important fact is that changes in lifestyle as a result of the confinement have led to unhealthy diet changes, as observed in other studies, due to emotional and uncontrollable eating to cope with the stress and anxiety caused by the uncertainties of the pandemic (Cecchetto et al., 2021; Vila‐Marti et al., 2022). Changes in eating behavior might also be related to access to food markets, already existing unhealthy eating behaviors prior to the pandemic, and mental health issues such as depression, anxiety, and stress, which can influence the behavior tendencies toward a healthier diet or an unhealthy diet (Madalı et al., 2021). Women were mostly affected during confinement periods, leading to mental health issues due to the elevated stress, unhealthy lifestyle, and diet (Mattioli et al., 2021).

In terms of PAL, the contrasting contexts of the two countries resulted in different outcomes. In RRM, there was a higher level of PAL and increased outdoor and indoor physical activities, with walking as the most popular activity among women and vigorous activities among men. This aligns with surveys on physical activity in Germany, where middle‐aged and older adults with a high level of education were particularly likely to increase physical activity during the pandemic, considering that the PAL was widespread in the population before the pandemic (Nowossadeck et al., 2021; RKI, 2021). However, a noticeable reduction in physical activity levels was self‐reported in the studied Brazilian regions. Self‐isolation and obligatory hygiene and social‐distancing measures, such as the use of masks and limited access to green urban spaces, could be the leading reasons behind the lower level of physical activity in both countries' contexts (Governo do Estado de São Paulo, 2021; Ribeiro de Lima et al., 2021; RKI, 2021; Ximenes & Maglio, 2020).

The sociodemographic characteristics were homogenous among the studied metropolitan regions – middle‐aged women, highly educated from the middle and upper classes, which needs to be considered when interpreting our study's results. This limitation, derived from the sampling strategy (convenience sampling) and the survey dissemination medium (online), implied that only participants with internet access had the opportunity to participate. Due to this selection bias, the sample cannot be considered representative but is skewed, as described above. Also, there was self‐selection bias, in which the participant felt attracted by the topic and intentionally decided to participate. However, achieving a representative sample constitutes a challenge per se, where the design‐based approach of random sampling is not always applicable (Zhao, 2021). Another limitation of the study is the use of FFQ as a tool for dietary assessment. The use of FFQ comes with recall bias and errors due to underestimation or overestimation of the quantity of food consumed (Popoola et al., 2023; Thieleking et al., 2023). Moreover, our intention was not to quantify food intake but to investigate the food consumption tendencies during the pandemic, for which FFQ can be a cost‐effective tool to collect data from a wide range of populations.

Strikingly, nearly 60% of the participants reported avoiding eating meat (or eating less) due to their enhanced awareness of the presumed zoonotic origin of the SARS‐CoV2 pathogen. This suggests interesting questions for further research on meat consumption practices, including whether such changes during the pandemic are sustained in post‐pandemic society. Further research on dietary and behavioral changes that remained after the pandemic is required to investigate whether changes were kept or were a phenomenon observed during the lockdown. Reproducing this study with a more representative sample within different population cohorts and socioeconomic strata would validate the outcomes. More detailed information on the reasons behind dietary and lifestyle changes in future studies would facilitate explaining the changes reported here.

## 5 CONCLUSION

This study investigates self‐reported changes in eating habits, buying groceries, physical activity, and other individual habits during the first year of the COVID‐19 pandemic across metropolitan regions in Brazil and Germany. Considering the homogeneity of the sample, with a high proportion of middle‐aged and highly educated females from the middle and upper classes in both countries, it is possible to infer that in Brazilian metropolitan regions, greater emphasis was given to healthy and sustainable eating and concerns related to hygiene measures and food delivery. In the German RRM metropolis, convenient food consumption increased as people avoided going to the supermarket, and the perception that supply was of lower quality was higher. An increase in physical exercise was observed in the German metropolis, while in Brazilian metropolitan regions, physical activity decreased. Our study is relevant to understanding better behavioral changes in different cultural and spatial contexts to cope with emergencies, raise awareness, and prepare for future pandemics.

## AUTHOR CONTRIBUTIONS

**Juliana M. G. Paris:** Conceptualization (equal); formal analysis (lead); investigation (lead); visualization (lead); writing – original draft (lead); writing – review and editing (lead). **Emília M. F. Lima:** Conceptualization (equal); investigation (equal); writing – original draft (equal); writing – review and editing (equal). **Jéssica de A. F. F. Finger:** Conceptualization (equal); investigation (equal); writing – original draft (equal); writing – review and editing (equal). **William R. Isidorio:** Conceptualization (equal); investigation (equal); writing – original draft (equal); writing – review and editing (equal). **Christine Heinzel:** Investigation (equal). **Timo Falkenberg:** Conceptualization (equal); funding acquisition (equal); writing – review and editing (equal). **Christian Borgemeister:** Conceptualization (equal); funding acquisition (equal); supervision (equal); writing – review and editing (equal). **Uelinton M. Pinto:** Conceptualization (equal); supervision (equal); writing – review and editing (equal). **Ute Nöthlings:** Conceptualization (equal); supervision (equal); writing – review and editing (equal).

## CONFLICT OF INTEREST STATEMENT

The authors declare that they do not have any conflicts of interest.

## ETHICAL STATEMENTS

This study was approved by the Committee of the Faculty of Pharmaceutical Sciences, University of São Paulo (CAAE 5331781720.9.0000.0067) and the Research Ethics Committee, Center for Development Research (ZEF), University of Bonn, Germany (13c_19).

## INFORMED CONSENT

Written digital informed consent was obtained from all study participants before the survey.

## Supporting information


Data S1.

Data S2.

Data S3.

Data S4.

## Data Availability

The data that support the findings of this study are available in the supplementary material of this article.
