# Supplementary material for: Changes in eating habits and lifestyle during the first year of the COVID‐19 pandemic across metropolitan regions in Brazil and Germany: A survey‐based cross‐sectional study
Source: Food Sci Nutr. 2024 Jan 22;12(4):2783–98. doi: 10.1002/fsn3.3960 (PMC11016392; doi:10.1002/fsn3.3960)
Supplement: Supplementary file 4 — Data S4. [file FSN3-12-2783-s004.docx]

**Supplementary Information S4**

**Table S1. Healthy Eating Index components and scoring criteria applied (0 to 1).**

| **Components** | **Scoring Criteria** |
| --- | --- |
| **Cereals, tubers, and roots** | < median 0  ≥ median 1 |
| **Fruit** | < median 0  ≥ median 1 |
| **Vegetables** | < median 0  ≥ median 1 |
| **Meat and eggs** | < median 1  ≥ median 0 |
| **Fish and seafood** | < median 0  ≥ median 1 |
| **Milk and milk products** | < median 0  ≥ median 1 |
| **Pulses and oilseeds** | < median 0  ≥ median 1 |
| **Oils and fats** | < median 1  ≥ median 0 |
| **Sugar and sweets** | < median 1  ≥ median 0 |
| **Beverages** | For coffee, tea, and water  < median 0  ≥ median 1  For soft drinks and juices  < median 1  ≥ median 0 |
| **Ultra-processed foods** | < median 1  ≥ median 0 |
| **Alcohol** | Men 1  Women 1 |


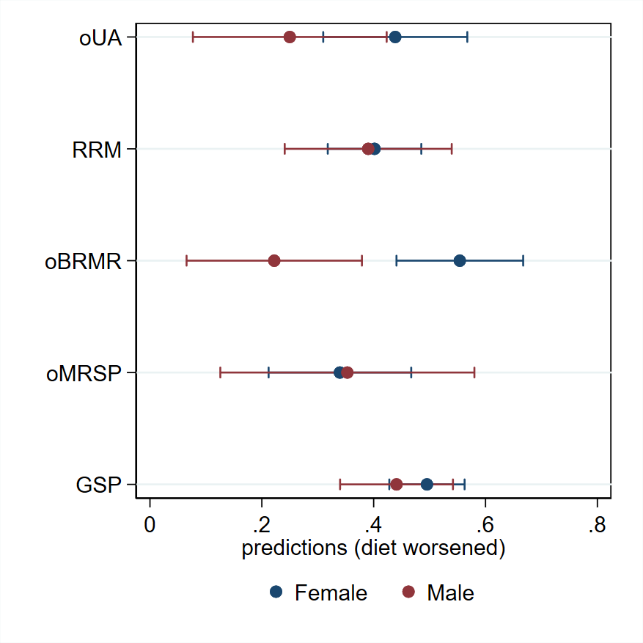

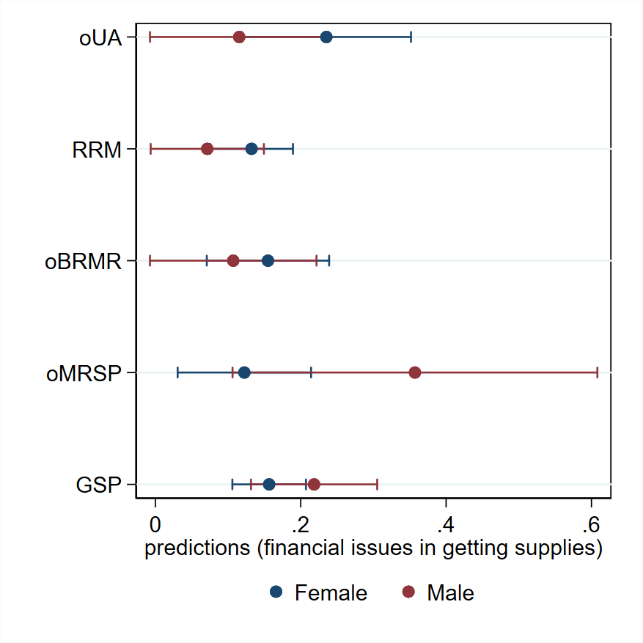

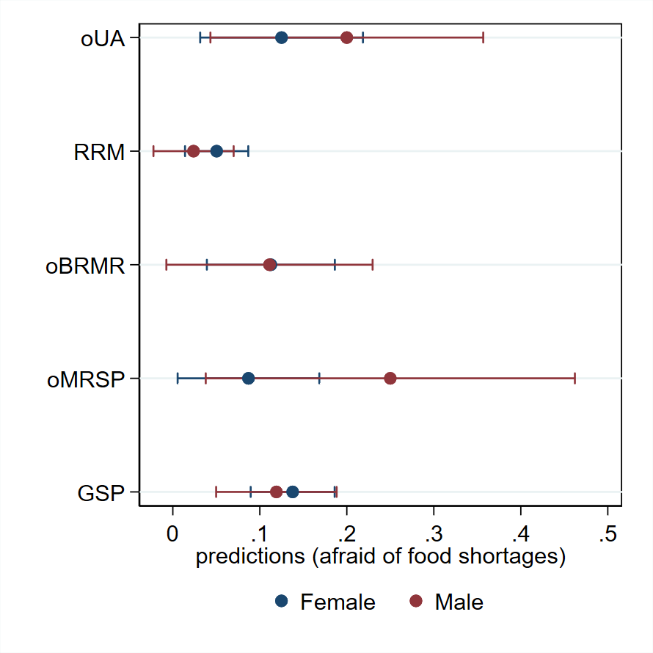

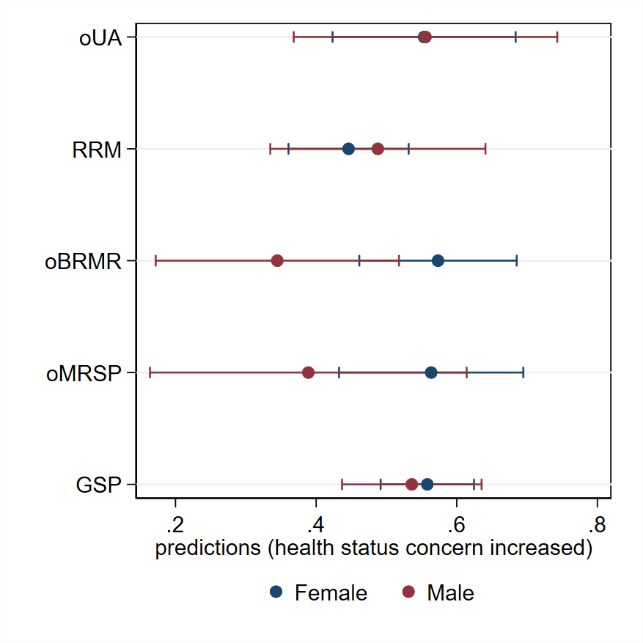

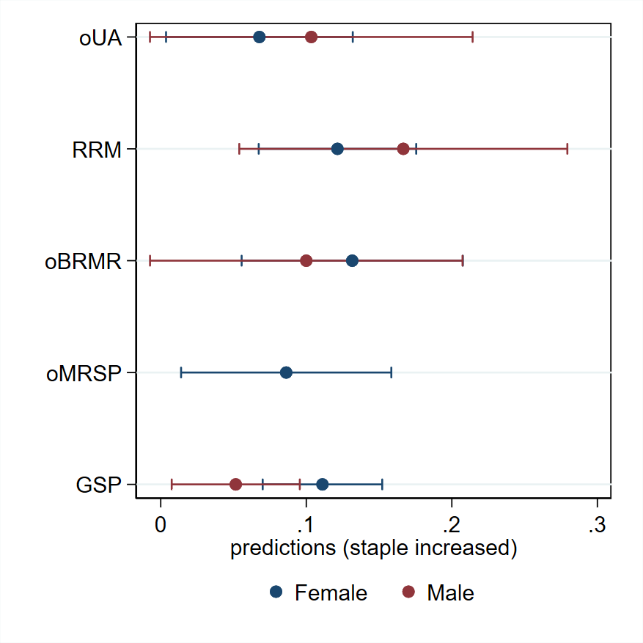


**Figure S4. Supplementary results on self-reported changes in eating habits and buying groceries between sex and the different metropolitan regions**. Dots represent adjusted predictions from no change (0) to change (1) of the logistic regression correlations between sex and metropolitan areas. Error bars represent the 95% confidence intervals. Legend: GSP: Great São Paulo; oMRSP: other metropolitan regions in São Paulo state; oBRMR: other Brazilian metropolitan regions; RRM: Rhine-Ruhr Metropolis; oUA: other urban areas
